# Simple, Effective and Validated. VTE CASE Risk Assessment Score for Venous Thromboembolism in Metastatic Germ Cell Tumour Patients Before First‐Line Chemotherapy

**DOI:** 10.1002/cam4.70295

**Published:** 2024-10-10

**Authors:** Wojciech Michalski, Anna Macios, Grażyna Poniatowska, Inga Zastawna, Tomasz Demkow, Paweł Wiechno

**Affiliations:** ^1^ Department of Urological Cancer Maria Skłodowska‐Curie National Research Institute of Oncology Warsaw Poland; ^2^ Department of Cancer Prevention Maria Skłodowska‐Curie National Research Institute of Oncology Warsaw Poland; ^3^ Centre of Clinical Cardiology and Rare Cardiovascular Diseases National Medical Institute of the Ministry of the Interior and Administration Warsaw Poland

**Keywords:** deep vein thrombosis, germ cell tumour, pulmonary embolism, risk assessment score, venous thromboembolism

## Abstract

**Background:**

Venous thromboembolism (VTE) may jeopardise excellent treatment results of germ cell tumours (GCT). We previously constructed a VTE risk score for GCT patients qualified for first‐line chemotherapy (CTH), including vein compression, clinical stage (CS) and haemoglobin concentration.

**Aim:**

Validating our score in a separate cohort and establishing the cut‐off point for the score. Re‐assessing the numerical score in the training cohort.

**Materials and Methods:**

We retrospectively analysed a new cohort of GCT patients staged IS–IIIC. Area under the curve of receiver‐operating characteristic (AUC‐ROC) was calculated for the developed score, Khorana Risk Score (KRS) and Padua Prediction Score (PPS). AUC‐ROC of the integer score was calculated for the training cohort. Cut‐off point was established by Youden's and Liu's indices.

**Results:**

Among 336 eligible patients in the validation cohort, VTE occurred in 41 (12.2%). AUC‐ROC for our score, KRS and PPS were 0.818 (95% confidence interval (CI): 0.746–0.891), 0.608 (0.529–0.688) and 0.634 (0.547–0.720), respectively, *p* < 0.001. The optimal cut‐off point for a low/high risk was 6 (≤ 6 vs. ≥ 7). In the training cohort, 369 patients had complete data on vein compression. AUC‐ROC for our score, KRS and PPS were 0.819 (95% CI: 0.758–0.879), 0.710 (0.637–0.782) and 0.725 (0.651–0.800), *p* ≤ 0.001 and 0.015, respectively. Positive and negative predictive values were 30.8% and 96.5%, respectively.

**Conclusions:**

Our VTE risk score is a handy tool for GCT patients before first‐line CTH for metastatic disease. Outperforming KRS and PPS, it has a good discriminatory value, especially for identifying low‐risk patients.

## 1 Background

Men diagnosed with metastatic germ cell tumours (GCT) are at a high risk of venous thromboembolism (VTE) [1, 2, 3, 4, 5]. The first 3 months from diagnosis or anticancer treatment initiation carry the highest VTE risk (> 50% of VTE cases) [6]. In the first year after BEP (bleomycin, etoposide and cisplatin) chemotherapy (CTH), VTE risk increases over 20‐fold and decreases over time (hazard ratio, HR 24.7 in the first year and 1.4 after 10 years) [7, 8]. A significant percentage of VTE cases occur prior to CTH [1]; still, cisplatin increases the risk even further (relative risk, RR 1.67; 95% confidence interval (CI): 1.25–2.23; *p* = 0.01), especially for equivalent weekly dose > 30 mg/m^2^ (RR 2.71; 95% CI: 1.17–6.30; *p* = 0.02) [9]. According to some studies, VTE correlates with overall survival (OS) [10, 11], hence deteriorating the excellent curability of GCT. Selecting patients at risk and applying thromboprophylaxis is therefore of paramount importance.

European Society for Clinical Oncology (ESMO) recommends considering thromboprophylaxis in metastatic GCT patients receiving cisplatin‐based CTH, especially with retroperitoneal lymph nodes (RPLN) > 3.5 cm, clinical stage (CS) III or poor‐risk features [8]. Furthermore, thromboprophylaxis is indicated in hospitalised patients confined to bed with acute medical conditions [6]. National Comprehensive Cancer Network (NCCN) recommends considering thromboprophylaxis for Khorana Risk Score (KRS) ≥ 2, up to 6 months or longer, if the risk persists [12]. Traditionally, risk assessment has been based mainly on KRS; however, risk factors specific for GCT, such as CS, RPLN or venous access device (VAD), may additionally define groups bearing a higher VTE risk [8].

In our previous study [13], we analysed a broad panel of clinical, pathological and laboratory parameters potentially correlating with VTE risk in GCT patients. VTE incidence was 13.9% [13], in line with published research [1, 2, 3, 4, 5]. In 58.8% of the patients, VTE occurred prior to first‐line chemotherapy [13], which underpinned the importance of disease‐related factors in VTE pathophysiology. A multi‐parametric risk assessment model (RAM) was constructed and checked for discriminatory performance and goodness of fit. Area under the curve of receiver‐operating characteristic (AUC‐ROC) for our model, KRS and Padua Prediction Score (PPS) were 0.885, 0.588 and 0.700, respectively [13]. Vein compression on computed tomography (CT), CS IIIB–IIIC and a decrease in haemoglobin (Hgb) concentration (with assigned weights of 7, 4 and 1 for 1 g/dL decrease, respectively) were included in a three‐item risk score for VTE (throughout this paper referred to as the ‘modelled score’) in GCT patients before first‐line CTH.

## 2 Aim

The primary objective of this study was to validate our modelled score (described in the original paper) [13] in a new cohort of GCT patients at the start of the first‐line CTH. As before, we aimed to compare our score's performance with KRS and PPS.

Secondary objectives included analyses of VTE incidence and clinical presentation in the validation cohort. We also planned to evaluate the modelled integer score again in the training cohort, since in the previous study, we only analysed the 7‐item multivariate model from which the score was derived.

Due to the retrospective nature of our studies, varying policies of primary thromboprophylaxis, and the fact that the majority of VTE cases were diagnosed prior to chemotherapy administration, we were unable to focus solely on patients without prophylaxis and on‐treatment VTE. Therefore, our score was intended to highlight a ‘VTE high‐risk profile’ in patients first diagnosed with metastatic GCTs rather than quantify VTE risk attributed to chemotherapy itself.

## 3 Materials and Methods

### 3.1 Patients' Selection

We retrospectively searched our clinical databases for male patients with gonadal and extragonadal (retroperitoneal, mediastinal or intracranial) GCT (International Classification of Diseases, ICD‐10 codes: C62, C48, C38 and C71, respectively) who had undergone first‐line CTH at our institution in the years 2009–2010 and 2020–2023. We applied identical inclusion criteria and coding as in our previous study [13]: ‘Patients with disseminated disease, i.e. CS IS–IIIC according to Tumour—Node—Metastasis (TNM) classification (eighth edition), who commenced multiagent chemotherapy or at least prephase (if applied) were eligible. Of note, TNM classifications for primary retroperitoneal (C48) and mediastinal (C38) tumours are designed mainly for soft tissue sarcomas. Moreover, tumours of the central nervous system (C71) are not routinely staged with TNM system; their dissemination is defined as radiological lesions along the cerebrospinal axis or positive cerebrospinal fluid cytology. For the sake of this study, we adapted TNM used for testicular GCT (C62) to describe extragonadal GCT as well (i.e. T0 N0–3 M0–1 S0–3) to maintain consistent staging’.

Stage IS is defined as any T, N0, M0, S1–S3 (all the following: any primary tumour classification, absence of regional nodal and distant metastatic involvement on CT and abnormal serum marker concentrations, persisting or increasing after orchiectomy). In higher stages (II–III), dissemination is visible on CT, involving regional or distant lymph nodes or visceral metastases, or both, with or without elevated serum marker concentrations [14].

Additionally, we re‐analysed the training cohort, described in detail in the previous paper [13].

The study was approved by the Ethical Committee at Maria Skłodowska‐Curie National Research Institute of Oncology, Warsaw, Poland (consent no. 28/2024; 21 March 2024).

### 3.2 Materials

The following data were extracted for the validation cohort:

- demographics: age, body mass, height, body mass index (BMI).
- disease‐related: CS, International Germ Cell Cancer Collaborative Group (IGCCCG) prognosis, histology (seminoma vs. non‐seminoma), localisation of the primary tumour (gonadal vs. extragonadal), vein compression.
- treatment‐related: extended (between CTH cycles) VTE prophylaxis.
- laboratory: Hgb, platelet (Plt) and leukocyte (WBC) count before CTH start.
- endpoint: overt deep vein thrombosis (DVT) and/or pulmonary embolism (PE); time (days) from CTH start to the event.

We only were able to identify vein compression in radiological CT reports, not on the scans themselves. We searched for expressions like ‘vein compression’, ‘vein narrowed’, ‘slowed venous flow’ or anything to that effect.

Venous thromboembolism risk score was calculated according to KRS, PPS and the modelled score.

Identically, as in our previous study [13], ‘overt VTE was reported on

- CT, performed routinely for staging or follow‐up purposes (in asymptomatic cases).
- Doppler ultrasound, performed when DVT was suspected clinically.
- CT angiography, performed when PE was suspected clinically.
- in medical records from the follow‐up period.

For the reasons mentioned in the ‘Aim’ section, we included all VTE cases from the diagnosis of metastatic GCT (occurring prior to or during chemotherapy). The medical records were screened for VTE up to 6 months after the end of first‐line chemotherapy or until residual lesions surgery, second‐line CTH, any radiotherapy or patient's death, whichever occurred first’.

The original training cohort was narrowed down to patients with complete data regarding vein compression (CS and Hgb prior to CTH were available for all patients). VTE risk score was calculated according to KRS, PPS and the modelled score.

### 3.3 Statistics

Stata15.1 software was used. According to the results of our original study, we assumed VTE incidence to be 13.0%–14.0%. The expected AUC‐ROCs of our model and comparator models were 0.800 and 0.700, respectively. The minimal number of observations required for validation was calculated: 233 (including 29 VTE events) or 320 (including 39 VTE events) for tests' power of 0.8 or 0.9, respectively.

Clinical, histological and laboratory parameters were compared between two subgroups, that is, patients with and without VTE, VTE (+) and VTE (−), with significance threshold of 0.05.

Ordinal variables were compared with the chi‐squared test; continuous variables were first checked for normality of distribution with Shapiro–Wilk test. Further comparisons were conducted with Student's *t* test or Mann–Whitney–Wilcoxon test, for variables with normal or non‐normal distributions, respectively.

Continuous variables were presented as means and standard deviations in case of normal distribution and as medians and interquartile ranges (IQR) in case of non‐normal distribution; discrete variables—as numbers and percentages.

Descriptive statistics were presented for VTE incidence, its location (DVT, PE and veins involved), timing (prior to vs. on CTH) and occurrence in patients with or without extended prophylaxis with low molecular weight heparin (LMWH).

Our risk score was compared to KRS and PPS in terms of AUC‐ROC statistics. We applied the following interpretation of AUC predictive values: 0.5—random, 0.5–0.7—low, 0.7–0.8—acceptable, 0.8–0.9—good/very good, > 0.9—excellent [15].

Additionally, we calculated the AUC‐ROC of the modelled integer score as well as the 3‐item logistic model for the narrowed training cohort, as described in the ‘Materials’ section (in the previous study, we only analysed the 7‐item model from which the score was derived).

As a *post hoc* analysis, we re‐assigned integers to our score according to the method first described by Schneeweiss et al., based on the increase in beta coefficients. Scoring weights here increase by 1 unit with each 0.3 increase in the beta; therefore, the weight of 1 refers to an e^(0.30)^ = 35% risk increase [16]. The new AUC score was compared to our original score as well as to KRS and PPS.

The cut‐off point was determined according to Youden's and Liu's indices in both the validation and training cohort.

Finally, since vein compression was the strongest VTE predictor (OR 8.96 in the training cohort [13]), and only minority of high‐risk patients presented with no compression, we decided to test vein compression as a single predictor against the modelled 3‐item score.

## 4 Results

### 4.1 Patients' Characteristics

We identified 336 patients fulfilling the inclusion criteria for the validation cohort. Detailed data for subgroups with and without VTE diagnosis are presented in Table 1.

**TABLE 1:** Validation cohort—patients' characteristics.

Variable	VTE (+), *n* = 41	VTE (−), *n* = 295	*p*
Age, median (IQR)	31 (11)	33 (16)	0.273
BMI, median (IQR)	25 (5.1)	25.9 (5.1)	0.440
Hgb, median (IQR)	15 (1.7)	13.2 (2.9)	< 0.001
Plt, median (IQR)	256 (89)	279 (162)	0.058
WBC, median (IQR)	6.7 (2.8)	7.1 (3.0)	0.133
Histopathology, *n* (%)			
Seminoma	16 (39)	83 (28.1)	0.152
Non‐seminoma	25 (61)	212 (71.9)	
Primary tumour, *n* (%)			
Testicular	33 (80.5)	276 (93.6)	0.004
Extragonadal	8 (19.5)	19 (6.4)	
CS, *n* (%)			
IS–IIIA	11 (26.8)	204 (69.2)	< 0.001
IIIB–IIIC	30 (73.2)	91 (30.8)	
IGCCCG prognostic group, *n* (%)			
Favourable	18 (43.9)	214 (72.5)	< 0.001
Intermediate	9 (22)	41 (13.9)	
Poor	14 (34.1)	40 (13.6)	
Vascular compression, *n* (%)	33 (80.5)	64 (21.7)	< 0.001
LMWH extended prophylaxis, *n* (%)	11 (26.8)	170 (57.6)	< 0.001
KRS, *n* (%)			
1–2	38 (92.7)	276 (93.6)	0.832
> 2	3 (7.3)	19 (6.4)	
PPS, *n* (%)			
3	12 (29.3)	155 (52.5)	0.013
4–5	22 (53.7)	115 (39.0)	
> 5	7 (17.1)	25 (8.5)	
Modelled score, median (IQR)	7 (6)	0 (12)	< 0.001

Abbreviations: BMI, body mass index; CS, clinical stage; Hgb, haemoglobin concentration; IGCCCG, International Germ Cell Cancer Collaborative Group; IQR, interquartile range; KRS, Khorana Risk Score; LMWH, low molecular weight heparin; *n*, number; Plt, platelet count; PPS, Padua Prediction Score; VTE, venous thromboembolism; WBC, white blood count.

Of 495 patients in the training cohort, 369 had complete data regarding vein compression; CS and Hgb were available for all patients.

### 4.2 VTE Incidence and Clinical Presentation

In the validation cohort, VTE was diagnosed in 41 patients (12.2%). Of the VTE (+) group, DVT alone occurred in 26 (63.4%), PE alone in eight (19.5%), and both presentations in seven (17.1%) patients—synchronically in five (71.4%) and metachronically in two (28.6%) cases.

In 15 patients (36.6% of the VTE group), DVT was seen in more than one vein. The most frequent locations were inferior vena cava—11 cases (26.8% of the whole VTE group), common iliac—11 (26.8%), renal—eight (19.5%), femoral—seven (17.1%) and external iliac veins—four (9.8%). Internal iliac veins accounted for two cases (4.9%). One (2.4%) case of DVT was diagnosed in each of the following veins: brachiocephalic, internal jugular, hepatic portal, popliteal sigmoid sinus and transverse sinus. No patient died due to VTE.

Venous thromboembolism occurred in 23 of 41 patients (56.1%) prior to CTH and in 18 (43.9%) after CTH started. In the latter group, the median time from CTH day 1 to the VTE event was 82 (5–158) days.

Of 155 patients not receiving extended LMWH prophylaxis, 30 (19.4%) experienced VTE. This subgroup, however, included patients with VTE diagnosed prior to chemotherapy (23 of 30; 76.7%). Of 181 patients with extended thromboprophylaxis during CTH, VTE was diagnosed in 11 (6.1%).

In the limited training cohort of 369, VTE was diagnosed in 51 patients (13.8%).

### 4.3 KRS and PPS in the Validation Cohort

According to KRS, 22 (6.5%) patients had high VTE risk (KRS ≥ 3) and the remaining 314 (93.5%) had intermediate risk. VTE incidence was 13.6% (3/22) and 12.1% (38/314) in the high‐risk and intermediate‐risk groups, respectively. Of all VTE events, 7.3% (3/41) occurred in high‐risk patients.

According to PPS, 32 (9.5%), 137 (40.8%) and 167 (49.7%) patients scored > 5, 4–5 and 3, respectively. VTE incidence in the subgroups was 21.9% (7/32), 16.1% (22/137) and 7.2% (12/167). Of all VTE events, 17.1% (7/41) and 53.7% (22/41) occurred in patients scoring > 5 and 4–5, respectively.

Receiver‐operating characteristic statistics favoured PPS over KRS pointwise, with an AUC of 0.634 (95% CI: 0.547–0.720) and 0.608 (0.529–0.688), respectively. However, the difference was insignificant (*p* = 0.638).

### 4.4 The Modelled Risk Score in the Validation Cohort

**FIGURE 1:**
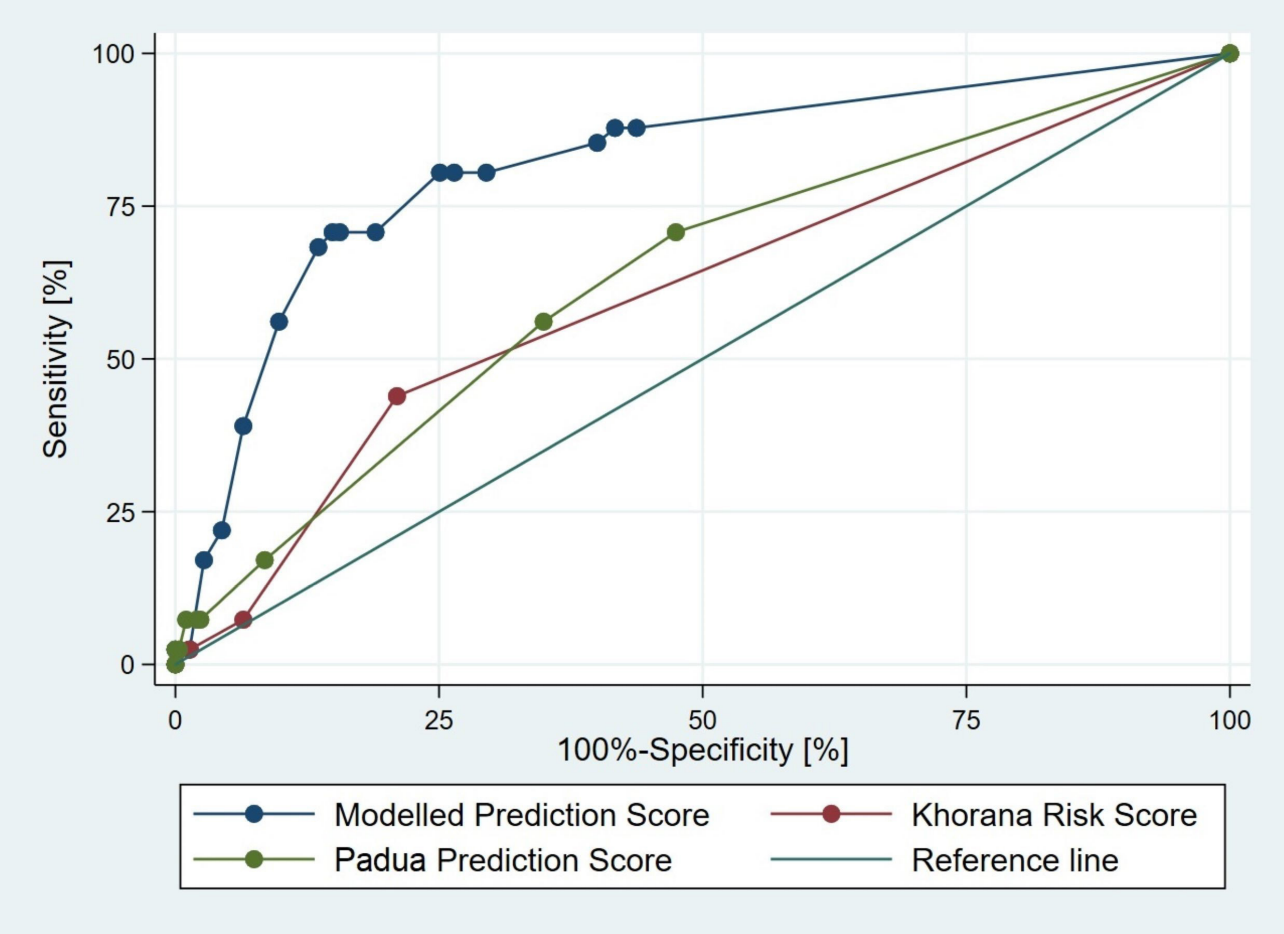
Receiver‐operating characteristics (ROC) curves for the modelled prediction score (blue line), Khorana Risk Score (red line) and Padua Prediction Score (green line) in the validation cohort.

According to ROC statistics, with an AUC of 0.818 (95% CI: 0.746–0.891), our risk score proved superior to both KRS and PPS pointwise (Table 2, Figure 1), and the difference between these three scores was significant (*p* < 0.001). In pairwise comparison of our risk score with KRS and with PPS, both differences were statistically significant (Table 2).

**TABLE 2:** Comparison of the modelled VTE risk score with KRS and PPS in the validation cohort.

VTE risk score	AUC (95% CI)	*p*	
		vs. KRS	vs. PPS
Modelled score	0.818 (0.746–0.891)	< 0.001	< 0.001
KRS	0.608 (0.529–0.688)	—	0.638
PPS	0.634 (0.547–0.720)	0.638	—

Abbreviations: AUC, area under curve; CI, confidence interval; KRS, Khorana Risk Score; PPS, Padua Prediction Score; VTE, venous thromboembolism.

The optimal cut‐off point in the validation cohort was 9.5 according to Youden's index, and 6.5 according to Liu's index. Therefore, the low‐risk group could be defined as scoring ≤ 9 or ≤ 6, respectively.

### 4.5 The Modelled Score, KRS and PPS in the Training Cohort

In the training cohort, AUC for the three investigated risk scores were as follows: 0.819 (95% CI: 0.758–0.879) for the modelled score, 0.710 (0.637–0.782) for KRS and 0.725 (0.651–0.800) for PPS. Comparison of all three risk scores yielded statistically significant differences (*p* = 0.001). Our score proved superior to both KRS and PPS (Table 3). In contrast, the difference between KRS and PPS was insignificant (*p* = 0.693).

**TABLE 3:** Comparison of the modelled VTE risk score with KRS and PPS in the training cohort.

VTE risk score	AUC (95% CI)	*p*	
		vs. KRS	vs. PPS
Modelled score	0.819 (0.758–0.879)	< 0.001	0.015
KRS	0.710 (0.637–0.782)	—	0.693
PPS	0.725 (0.651–0.800)	0.693	—

Abbreviations: AUC, area under curve; CI, confidence interval; KRS, Khorana Risk Score; PPS, Padua Prediction Score; VTE, venous thromboembolism.

AUC‐ROC for the multivariate model with the three variables (i.e., vein compression, CS III B–III C and Hgb) was 0.827 (95% CI: 0.769–0.885).

The optimal cut‐off point in the training cohort was 7.5 according to Youden's index, and 8.5 according to Liu's index. Therefore, the low‐risk group could be defined as scoring ≤ 7 or ≤ 8, respectively.

### 4.6 Schneeweiss Scoring System

According to the Schneeweiss scoring rule [16], vein compression and Hgb retained their weights of 7 and 1 per 1 g/dL decrease, respectively. CS IIIB–IIIC, in turn, scored 6 instead of 4. In the training cohort, the new score's AUC was 0.820 (95% CI: 0.760–0.880); the difference was insignificant with respect to our original score (*p* = 0.615), and significant with respect to KRS (*p* < 0.001) and PPS (*p* = 0.014). In the validation cohort, the new score's AUC was 0.815 (95% CI: 0.743–0.888); the difference was again insignificant with respect to our original score (*p* = 0.072) and significant with respect to KRS (*p* < 0.001) and PPS (*p* < 0.001). Therefore, we decided to maintain our scoring, as previously described [13].

### 4.7 Cut‐Off Point

We decided on the lowest possible cut‐off point for our score (6, according to Liu's index in the validation cohort) as the most relevant in clinical practice, for reasons described in the ‘Discussion’ section. Hence, the low risk and high risk for VTE development were defined as score ≤ 6 and ≥ 7, respectively. Sensitivity, specificity, accuracy, positive and negative predictive values (PPV and NPV) for both cohorts are presented in Table 4. The final version of the score is shown in Table 5.

**TABLE 4:** Diagnostic parameters of the modelled risk score in the training and validation cohorts, with the cut‐off point of 6 (≤ 6 vs. > 6).

Parameter	Training cohort (*n* = 369)	Validation cohort (*n* = 336)
VTE incidence (%)		
Total	13.8	12.2
High‐risk group (≥ 7)	29.2	30.8
Low‐risk group (≤ 6)	4.0	3.5
PPV (%)	29.2	30.8
NPV (%)	96.0	96.5
Sensitivity (%)	82.4	80.5
Specificity (%)	67.9	74.9
Accuracy (%)	69.9	75.6

Abbreviations: NPV, negative predictive value; PPV, positive predictive value; VTE, venous thromboembolism.

**TABLE 5:** VTE CASE score for metastatic germ cell cancer patients before first‐line chemotherapy.

Risk factor	Score
Vein compression (CT)	7
Clinical stage IIIB–IIIC	4
Haemoglobin concentration^a^	1 for every 1 g/dL

*Note:* VTE risk: ≤ 6—low; ≥ 7—high.

Abbreviations: CASE, *C*ompression, Anaemia and Stage Effect; CT, computed tomography; Hgb, haemoglobin; LLN, lower limit of normal; VTE, venous thromboembolism.

^a^ Only if below the LLN: the difference between LLN and patient's concentration, LLN—[Hgb], rounded up to an integer.

### 4.8 The Modelled 3‐Item Score Versus Vein Compression Alone

Among high‐risk patients (scoring ≥ 7), those without vein compression accounted for 3% (4/144) in the training cohort and 9% (10/107) in the validation cohort. Therefore, we performed additional analyses assessing the predictive accuracy of sole vein compression. In the training cohort, vein compression alone performed significantly worse than the 3‐item score (AUC‐ROC 0.746 vs. 0.819, respectively, *p* < 0.001) and similar to KRS and PPS (*p* = 0.304 and 0.603, respectively). In the validation cohort, contrarily, vein compression alone performed similar to the 3‐item score (AUC‐ROC 0.794 vs. 0.818, respectively, *p* = 0.146) and significantly better than KRS and PPS (*p* < 0.001 and *p* = 0.003, respectively).

## 5 Discussion

Contemporarily, there has been a trend towards personalised oncology: individual prognoses and mutation‐driven therapeutic agents. A similar tendency may be noticed in supportive care, venous thromboprophylaxis included. The diversity of cancer types and their biology as well as different procoagulant properties of various medications necessitate more differentiated RAMs; a ‘one‐size‐fits‐all’ approach is no longer justified [6]. KRS has been the most validated model since 2008; however, most VTE cases occur in the low‐ and intermediate‐risk groups [17]. Subsequent attempts to improve KRS led to derivation of new scores, applicable in various settings: Vienna CATS, PROTECHT, CONKO004, ONCOTEV, COMPASS‐CAT, Tic‐ONCO or MDACC CAT [17]. Newest models have addressed specific cancer types, such as gastrointestinal [18, 19, 20], lung cancer [21, 22], lymphomas [23], multiple myeloma [24], acute lymphoblastic leukaemia [25] or particular clinical situations, for example, breast cancer patients with peripherally inserted central catheters [26]. Some scores include types of therapy, such as hormonal [19, 26, 27, 28], immunomodulatory drugs [24] or CTH [19, 22]. Finally, modern scores will incorporate particular mutations [22], microRNA [29] or machine learning [20].

In pursuit of an efficient RAM for GCT patients, various factors have been found to significantly correlate with VTE risk, for example, CS [4, 10, 30, 31, 32, 33], retroperitoneal primary [2], age [34], body surface area (BSA) [35], RPLN [1, 2, 3, 5, 31, 33, 36, 37], elevated lactate dehydrogenase (LDH) [1, 10, 35, 36], central venous catheter [1, 5, 10, 32] or access device [2, 38], number of CTH cycles [39], febrile neutropenia [10], absolute neutrophil count (ANC) [37], albumin [37], C‐reactive protein (CRP) [5] or baseline factor VIII concentrations [40]. Some studies indicated RPLN > 5 cm as the cut‐off diameter [3, 5, 33], whereas other researchers proved RPLN > 3.5 cm to be a more accurate VTE predictor [2].

Receiver‐operating characteristic statistics were calculated in three studies involving GCT patients [2, 3, 31]. Srikanthan et al. found that RPLN > 5 cm had higher discriminatory accuracy for VTE risk than KRS (AUC 0.71 vs. 0.67, respectively, in the training cohort, and 0.61 vs. 0.57 in the validation cohort) [3]. CS, in turn, as stratified by Bezan et al. [31] performed better than RPLN alone (AUC 0.75 vs. 0.63, respectively; *p* = 0.007 in the training cohort and 0.88 vs. 0.76; *p* = 0.04 in the validation cohort). However, in the metastatic setting, the differences between the two models were insignificant [31]. Finally, Tran et al. reported on AUC 0.632; *p* < 0.001 for RPLN > 3.5 cm as a single predictor [2]. Against this background, our score presents a very good discriminatory value of AUC 0.819 and 0.818 in the training and validation cohorts, respectively, with KRS and PPS performing significantly worse.

A few aspects of our risk score need to be further discussed. Pre‐CTH VTE in GCT patients may be a different entity from VTE occurring during or after CTH. The former seems to occur more often in men with GCT of the right testicle, with higher LDH, RPLN > 5 cm and KRS ≥ 3, whereas patients presenting with on‐CTH VTE are more like those without VTE [41]. Therefore, most authors excluded patients with pre‐chemotherapy VTE from their analyses of risk factors [2, 4, 5, 10, 31, 35, 36], whereas some did not [32]. In our cohorts, we analysed all patients together; nevertheless, the narrowed approach seems auspicious and should be further investigated. The ideal approach would be excluding patients with pre‐chemotherapy VTE and those on primary thromboprophylaxis; this would leave a uniform group to assess risk factors for chemotherapy‐associated VTE. However, this would limit the study group to highly selected patients with low‐stage, low‐volume disease, as indications for primary thromboprophylaxis have become more extensive over the last years. According to ESMO, ‘prophylaxis of thromboembolic events should be considered in metastatic germ cell tumour patients for the chemotherapy duration, especially when presenting with one or more of the established risk factors: retroperitoneal lymph nodes > 3.5 cm, stage III disease, central venous access catheter, intermediate or poor‐risk features or immobilisation’ [8]. Therefore, the group without primary prophylaxis would only include patients with clinical stage IS (with no radiologically visible metastatic lesions), IIA (retroperitoneal lymph nodes ≤ 2 cm) and, partially, IIB (retroperitoneal lymph nodes 2–3.5 cm among 2–5 cm in all CS II B patients). Such study design would identify new risk factors (e.g., pathological, laboratory, chemotherapy‐related, etc.) in those seemingly ‘healthy’ individuals; this would possibly lead to new indications for thromboprophylaxis. On the other hand, excluding patients with pre‐CTH VTE might result in the loss of valuable information contributing to the characteristic of a ‘VTE‐prone GCT patient’. The fact that VTE has already occurred does not negate the findings; a hypothetical patient not diagnosed with VTE at the start but fitting this profile should certainly be offered primary thromboprophylaxis. Such an approach was adapted by Dieckmann et al. [32]—patients with VTE at diagnosis (30%) were included as ‘providing more evidence for the contribution of disease‐related factors for the development of thromboembolic events’.

In our present cohorts, after excluding patients with VTE at diagnosis and those with primary thromboprophylaxis, the number of VTE events would be 14 (seven in the training and seven in the validation group). This, unfortunately, would generate insufficient tests' power and not allow to draw relevant conclusions.

A related issue is the correct assessment of VTE timing. Some patients had their baseline CT performed at the time of orchiectomy, some later, just before starting chemotherapy. It is not impossible that in some of the former group, VTE actually occurred prior to chemotherapy, without being suspected until patients presented with symptoms or even until the post‐chemotherapy CT re‐staging. Retrospective studies, without pre‐planned CT timing, cannot capture those events correctly. Honecker et al. [1] recorded as many as 81% VTE cases prior to chemotherapy and speculated that ‘a number of the seemingly therapy‐associated VTE events had in fact already been pre‐existent before the initiation of chemotherapy’.

Computed tomography scans were performed at our Institute or elsewhere. Retrospective assessment of vein compression, based solely on radiological reports, may cause a further bias. We imagine that in case of massive retroperitoneal or mediastinal involvement, the compression is apparent and unmistakably reported. However, unfortunate localisation of smaller lesions may also possibly cause such compression. Ideally, staging CT scans should be prospectively assessed and compressed veins specifically looked for.

We present an integer score, which seems convenient and practical. However, from the statistical point of view, each step of transforming multivariate regression models into integer scores may lead to a decrease in prediction accuracy. Hence, regression models and numerical scores often present with different statistical metrics.

As described in the original paper [13], we retained Hgb as a continuous variable, since our attempts to convert it into a categorical (binary) variable with different cut‐off points resulted in lower AUC‐ROCs. Indeed, it is due to dichotomising continuous variables that prediction models usually lose most of their original accuracy [42]. Still, our approach lies somewhere in the middle. The correlation of Hgb and VTE risk turned out to be nearly linear [13]; yet we decided not to ‘penalise’ values remaining within the reference range (14.0–18.0 g/dL), even though the difference between two ‘normal’ Hgb concentrations might reach up to 4 g/dL. From the medical point of view, all blood count parameters may vary between healthy individuals. Moreover, Hgb exceeding normal limits is bound to have a contrary (procoagulant) effect, which we did not capture in our calculations. This is why in our score points are only assigned for anaemia (1 point for every 1 g/dL below the normal range).

Another drop in a model's accuracy may occur at the stage of rounding coefficients to integers but this usually results in a much smaller, even negligible error [42]. We must additionally keep in mind that in our score, Hgb difference (lower reference value minus a patient's result) is also rounded up.

Finally, there is a question of retaining or removing insignificant variables in a model. We decided to do the latter, hence reduce the score to three items; however, it is true that insignificant variables may adjust or control the model's stability in a way. On the other hand, even stepwise selection may decrease a model's accuracy through increased overfitting [42].

For these reasons, we decided to re‐analyse the training cohort and evaluate the statistical performance of the modelled integer score (in addition to the complete model [13]). The original training cohort of 495 patients had to be narrowed down to 369 due to missing data regarding vein compression; this was also a potential cause of bias. Furthermore, we calculated AUC for a hypothetical regression model comprising the three items used in our score. As it might have been expected, the shortened 3‐item model had a lower AUC‐ROC than the complete model (0.827 vs. 0.885, respectively; ∆ = 0.058). Fortunately, the loss on the transition from the model to the integer score was small (AUC 0.827 vs. 0.819, respectively; ∆ = 0.008).

Four values of the optimal cut‐off point were obtained, in both the validation and training cohorts, with Youden's and Liu's index for each cohort. Based on the subject–matter–knowledge approach, we assumed the lowest possible value (≤ 6 for the low‐risk group) as the most appropriate. All other values for the low‐risk group (Youden's ≤ 9 and ≤ 7 or Liu's ≤ 8) would classify patients with vein compression (scoring 7) as having a low VTE risk. With inferior vena cava being compressed the most frequently, and the compression being the strongest VTE predictor, such approach would be misleading as those patients should require primary thromboprophylaxis when starting CTH.

Studies on thromboprophylaxis in GCT patients have given contradictory results. According to some authors, it decreased VTE incidence nearly by half [8, 33, 36]; others found it insignificant [5, 43]. Most authors did not exclude patients on primary thromboprophylaxis [1, 4, 10, 32, 33, 35], and its rate ranged from 9% [1] to 93% [10]. In our previous study [13], we found thromboprophylaxis significant in univariate analysis. Obviously, in clinical practice, LMWH prophylaxis only matters to those without VTE at the start of chemotherapy; for the remaining ones, it is too late. We decided to control this confounding effect in a statistical way—to ensure that potential risk factors in multivariate analyses were adjusted for that significant heparin effect, we entered the variable ‘LMWH prophylaxis’ into all of the six prediction models. But at this point, there are some misleading conceptions about this variable that come into the picture. First of all, it is very tempting to look upon LMWH prophylaxis as an intervention, in the way it would be managed in a prospective study in patients not presenting with VTE at diagnosis. Taking this approach further will lead us to a ‘catch 22’ situation—purportedly, administering (or not) LMWH prophylaxis affects the study's end point (i.e., identification of VTE risk factors), which, in turn, are potential new indications for VTE prophylaxis! This idea is only partially justified; it is true that our aim of finding additional VTE risk factors specific for GCT may, in the long run, result in offering thromboprophylaxis to additional patients commencing first‐line chemotherapy. However, we never aimed to assess LMWH prophylaxis as an intervention, with all the necessary parameters such as the numbers needed to treat and to harm, etc. In our study, LMWH prophylaxis served as an extraneous variable, with its potential effect of modulating the dependent variable (i.e., preventing, to some extent, VTE events). Yet, we were not interested so much in the magnitude of the effect itself as in whether LMWH would diminish the significance of other explanatory variables. That being said, the reason some patients had not been administered LMWH prophylaxis became less important. Some patients had their VTE diagnosed at the initial presentation and never had the chance to receive primary prophylaxis, some were assessed by their physician as having a low enough VTE risk to be exempted from prophylaxis—regardless of the reason, heparin did not affect the end point nor did it dilute the effect of other variables of interest. Factors from the best model in terms of predictive value and goodness of fit were then incorporated into the risk score.

Beneficial as thromboprophylaxis may be, the associated bleeding risk, possibly exacerbated by anticoagulation [5], is the other side of the coin. However, how bleeding should be evaluated in cancer patients is still an open question [44]. CAT‐BLEED, a recently developed RAM, counts genitourinary cancer, the association of gastrointestinal cancer with edoxaban and anticancer therapy with gastrointestinal toxicity as significant risk factors for bleeding in anticoagulated patients [44, 45]. With moderate c‐statistics of 0.61, it has still surpassed other existing bleeding risk scores, usually based on populations with a meagre number of patients with active cancer [44, 45, 46].

The limitation of our score, as well as many other RAMs, is its relevance at one time point, that is, prior to first‐line CTH. Meanwhile, Honecker et al. revealed different risk factors for VTE occurring prior to CTH (seminoma, retroperitoneal masses and elevated LDH) and on CTH (supraclavicular lymph node metastases and central venous catheters) [1]. Even after CTH start, VTE risk is not constant over the time of treatment; higher risk may be due to unplanned hospitalisations, periods of immobilisation, increasing cumulative dose of cisplatin (although not confirmed by some authors [1]), infections, granulocyte‐colony stimulating factor administration or other aspects. On the other hand, VTE risk may as well decrease within few days of the same hospitalisation [47]. Longitudinal assessment rather than a single observation of VTE‐associated parameters appears more useful—this has been proved for D‐dimer concentrations [48].

Bleeding risk may also change due to transient thrombocytopenia, changes in creatinine clearance, hepatic impairment, drug–drug interactions [44], massive dissemination of some non‐seminomatous components such as choriocarcinoma or rapid tumour lysis. Therefore, both VTE and bleeding risk should be assessed periodically throughout the course of CTH, presumably with different RAMs, to tailor thromboprophylaxis not only to an individual patient but also to a particular phase of treatment or clinical scenario.

Given the relatively low incidence of GCT, the main strength of our two studies is the number of patients included from one centre (831 overall in both cohorts). Apart from the already known VTE risk factors, that is, vein compression and higher clinical stage, we revealed that decrease in the baseline haemoglobin concentration correlated linearly with VTE risk. Therefore, the usual binary approach to anaemia in RAMs (most commonly < 10 g/dL or below normal) is more prone to assessment bias.

Moreover, the score is straightforward and practical. Its good predictive performance will certainly add to a better management of GCT patients in everyday practice.

## 6 Conclusions

Our risk score has proven to be of high discriminatory value for VTE prediction in metastatic GCT patients commencing first‐line CTH, superior to KRS and PPS. With the negative predictive value of 96.0%–96.5% (Table 4), it should help identify patients in whom primary extended thromboprophylaxis may possibly be safely omitted. However, we must keep in mind that different risk factors may contribute to on‐CTH VTE in patients with low‐stage, low‐volume disease; this remains to be elucidated in prospective, multi‐centre studies.

Obtaining a detailed CT report, with special attention paid to any vein compression, is crucial for the correct VTE risk assessment in GCT patients.

We have decided to name our modelled score VTE CASE, the acronym for ‘Venous Thromboembolism: Compression, Anaemia and Stage Effect’. All items are included here so that they can be easily memorised. Additionally, the acronym points to the general idea of the score, that is, presenting a characteristic of a GCT patient prone to VTE at diagnosis and throughout first‐line treatment.

Last but not least, we strongly encourage other researchers and oncologists dealing with GCT to use and validate our score in their own clinical practice; every feedback will be eagerly welcomed. We believe that it is in real life that the most valuable validation takes place.

## Author Contributions

**Wojciech Michalski:** conceptualization (lead), data curation (lead), formal analysis (lead), investigation (equal), methodology (equal), project administration (lead), supervision (lead), validation (lead), writing – original draft (lead), writing – review and editing (equal). **Anna Macios:** conceptualization (equal), formal analysis (equal), methodology (equal), software (lead), validation (equal), writing – review and editing (equal). **Grażyna Poniatowska:** data curation (supporting), formal analysis (supporting), writing – review and editing (equal). **Inga Zastawna:** conceptualization (supporting), formal analysis (supporting), writing – review and editing (equal). **Tomasz Demkow:** formal analysis (supporting), project administration (supporting), writing – review and editing (equal). **Paweł Wiechno:** formal analysis (supporting), project administration (supporting), writing – review and editing (equal).

## Ethics Statement

All procedures were performed in compliance with relevant laws and institutional guidelines. The study conforms to the Declaration of Helsinki and has been approved by the Ethics Committee at Maria Skłodowska‐Curie National Research Institute of Oncology, Warsaw, Poland (consent no. 28/2024; 21 March 2024). According to the Ethics Committee's decision, the study was exempt from obtaining written informed consent from patients. Only retrospective data were analysed. All prior medical procedures, examinations of biological materials, diagnostic imaging, pathology, radiology and laboratory reports as well as data from medical charts had constituted standard clinical management of germ cell tumour patients. No additional procedures were performed solely for the purpose of this study nor was any additional biological material obtained from the patients.

## Conflicts of Interest

The authors declare no conflicts of interest.

## Data Availability

The data that support the findings of this study are available upon request from the corresponding author. The data are not publicly available due to privacy or ethical restrictions.
